# Drivers of Utilization of HIV Self‐Test Among Males in Malawi: A Multilevel Analysis of the Multiple Indicator Cluster Survey 2019‐2020

**DOI:** 10.1155/arat/5527525

**Published:** 2025-11-09

**Authors:** Thokozani Mzumara, Yamikani Matewere, Mayamiko Mbamba, Chisomo White, Aaron Chidothe, Lazarus Obed Livingstone Banda, Grace Ogbonna, George Munthali, Rita Soko, Mlotha Mbughi, James Mkandawire

**Affiliations:** ^1^ Department of Curative and Rehabilitation Services, Mzuzu Community Hospital, Mzimba North District Health Office, Mzuzu, Malawi; ^2^ Key Population Programme, Mzimba North District Health Office, Mzuzu, Malawi; ^3^ Department of Optometry, Mzuzu University, Mzuzu, Malawi, mzuni.ac.mw; ^4^ Department of Humanities, Mchinji Mission Secondary School, Mchinji, Malawi; ^5^ Department of Computer Sciences, Nalikule College of Education, Lilongwe, Malawi; ^6^ Department of Finance, Mzuzu University, Mzuzu, Malawi, mzuni.ac.mw; ^7^ Department of Antiretroviral Therapy, Mzuzu Community Hospital, Mzimba North District Health Office, Mzuzu, Malawi; ^8^ Department of Laboratory Services, Mzuzu Community Hospital, Mzimba North District Health Office, Mzuzu, Malawi; ^9^ Directorate of Research, Invest in Knowledge, Zomba, Malawi

**Keywords:** HIV/AIDS, malawi, men, MICS, multilevel, regression, self-test, utilization

## Abstract

**Background:**

Implementation of health programs often overlooks males compared to females, and this is true even for HIV programs. Most men do not visit hospitals, and this is where, for example, most of the HIV services are delivered, including HIV testing. Self‐testing can help men know their HIV status and possibly prevent the spread and increase treatment uptake.

**Aim:**

This study aimed to assess the utilization of HIV self‐testing among males in Malawi and its predictors.

**Methods:**

This paper utilizes secondary quantitative data from the 2019‐2020 Multiple Indicator Cluster Survey (MICS). The study sample included 7750 males aged 15 to 49. Data analysis used the Chi‐square test to show the association between self‐testing and rural/urban residence, region, radio‐listenership, internet use, prior testing history, and educational level. Then, a multilevel binary logistic regression analysis was done to show the effect of predictor variables on the outcome reporting on the fixed and random effects, including the odds ratio, 95% confidence interval, and intraclass correlation coefficient. Statistical significance was determined at the *p* < 0.05 level.

**Results:**

Approximately 10% of males have ever used an HIV self‐test. The logistic regression model indicated that ever used the internet (OR = 1.535, CI: [1.231, 1.9150], *p* < 0.05), ever tested for HIV (OR = 0.181, CI: [0.122, 0.268], *p* < 0.001), rural residence (OR = 0.493, CI: [0.349, 0.698], *p* < 0.001), and region (OR = 3.619, CI: [2.120, 6.178], *p* < 0.001).

**Conclusion:**

The study confirms that HIVST is affected by several sociodemographic factors, calling for targeted interventions to improve HIVST among males.

## 1. Background

According to UNAIDS, approximately 40 million people were living with AIDS in 2023, and there were about 1.3 million people who became infected, a 64% reduction since the peak of 1995 [1]. However, the global burden disproportionately affects the sub‐Saharan region [2]. Early diagnosis remains key to improving health outcomes among people living HIV and is also critical to help curb its spread [3]. However, about 5 million people worldwide are still unaware of their HIV status and in need of HIV testing [3]. This is against achieving the public health goal of reaching 95% HIV status awareness levels by 2025.

In sub‐Saharan Africa, men, unlike women, are more likely to be unaware of their HIV status [4]. This can be explained by the exposure of women to HIV testing through antenatal services and, in part, can also be due to misguided masculinity norms in the sub‐Saharan region [4]. Previous research has indicated that a lack of knowledge of HIV status is a major limiting factor in participation in prevention and treatment programs. In sub‐Saharan Africa, the lesser involvement of men in HIV programmes has led to high mortality rates for the gender compared to women [5].

In general, men face unique challenges related to knowing their HIV status compared to women [6]. Accordingly, a study conducted in 2024 found that men are dissatisfied with general health services, which in turn affects HIV testing among men [7]. Qualities of the health system that negatively affect men include waiting times, costs, unavailability of medicines, and poor cleanliness. The advent of the HIV self‐test (HIVST) in 2000 was a significant milestone in the fight against HIV [3]. HIVST was introduced to address challenges caused by accessibility, stigma, and cost, which disproportionately affected vulnerable groups of the population, including men. Accordingly, men find HIVST appealing because of the autonomy, convenience, and confidentiality of the procedure [8].

The literature showcases a wide variation in the utilization of HIVST among men, echoing the different socioeconomic and cultural differences among geographical settings [9]. For instance, a study published in 2025 in Tanzania found that usage of HIVST among men was as low as 3.9% [3], but in Nigeria, it was reported at 97% [10]. In China, the prevalence of men using HIVST was estimated at 39%. The wide variation in utilization of HIVST is attributable to differences in cultures and lifestyle, beliefs, quality and availability of testing services, and knowledge regarding HIV across settings [6].

Although Malawi has made significant strides in the fight against HIV/AIDS [11], it remains one of Africa’s top 10 high–HIV‐burden countries [12]. A previous study conducted in 2018 among 4 districts in Malawi found that 45% of men have used HIVST [13]. Regardless, to the best of our knowledge, there is lack of nationally representative evidence on HIVST uptake among men in Malawi. Therefore, this paper aimed to assess the level of uptake and factors associated with the uptake of HIVST among men in Malawi. Policies and interventions developed based on nonrepresentative data may lead in ineffective and inequitable programs that fail to address the needs of underrepresented groups. The paper adopts the health belief model, which recognizes six constructs that influence health‐seeking behavior. Four out of six constructs focus on the aspect of perceptions and beliefs, while one focuses on self‐efficacy, and one dimension centers on cues to action. Specifically, this study focuses on the cues to action construct of the model to understand the factors that trigger testing among men [14]. The findings of this study will aid in planning policies and programs aimed at improving the uptake of HIVST in Malawi. Research suggests that more effort is needed to implement policies and programs that promote HIVST among men in sub‐Saharan Africa [4].

## 2. Methods

### 2.1. Design

This cross‐sectional quantitative study employed a quantitative approach using data from the Malawi Multiple Indicator Cluster Survey (MICS) 2019‐2020, accessed at https://mics.unicef.org/surveys. MICS provides up‐to‐date information on HIV, maternal health, childbearing, sexual behavior, child health indicators, fertility, sanitation, and hygiene. MICS is a nationally representative household survey. With technical support from UNICEF, Malawi’s National Statistics Office (NSO) and the Ministry of Health conduct MICS every four to 5 years. The study used the men’s dataset. The study included data from 7750 men.

### 2.2. Outcome Variable

The main outcome variable was “Have you ever used HIVST (Yes/No)?” and it was self‐reported and binary‐coded.

### 2.3. Independent Variables

#### 2.3.1. Individual‐Level Variables

Based on the literature, the study included the following variables: ever been tested, use of the internet, age, education, residence, and marital status [3].

#### 2.3.2. Contextual‐Level Variables

Location measured as rural or urban and region, including the north, central, and south, were the contextual‐level variables.

#### 2.3.3. Sampling Weights

The sample estimates from the MICS 2019‐2020 were applied to account for bias and generalization due to the nature of the MICS data.

### 2.4. Analysis

The data were analyzed using SPSS Version 27. The study used tables and graphs to illustrate the data graphically. For descriptive statistics, the review employed frequencies and percentages. For inferential statistics, the study employed the chi‐square test to assess the association between outcome and explanatory variables. Due to the hierarchical nature of the dataset, the study employed a multilevel binary logistic regression. Hence, the analysis accounted for clustering and corrects biased standard errors and shows cluster variability.

The multilevel regression analysis consisted of two models. Model 1 depicted the cluster variance without including the explanatory variables. Model 2 consisted of the individual‐level and contextual‐level variables. The model fit was assessed using the Akaike information criterion (AIC). Fixed and random effects were computed for all the models, and we reported fixed effects using odds ratio, *p* value, and 95% confidence interval, while random effects applied the intraclass correlation coefficient to assess the proportion of variance in the dependent variable due to group level differences. The value of *p* < 0.05 was considered statistically significant.

### 2.5. Ethics

Due to the nature of the study, ethical clearance was waived; however, the initial study was ethically approved and adhered to the Declaration of Helsinki. The study obtained permission to use the data set from UNICEF.

## 3. Results

After applying sampling weights, the proportions and inferential statisticcs will be reported with weighted estimates. As illustrated in Table 1, the majority, 6489 (95.6%) of 6791 participants, had ever attended school. About 676 (10.0%) had ever used HIVST kit. According to the area of residence, 5498 (81%) participants resided in rural areas. Based on the region of study, 2805 (41.3%) reside in the southern region, while 785 (11.6%) live in the northern region. According to the use of the internet, 1261 (18.6%) participants had ever used the internet. And 5728 (84.4%) listen to the radio.

**Table 1:** Characteristics of study variables.

Variable	Category	Frequency	Percent (%)
Ever used HIV self‐test kits	Yes	676	10.0
	No	6115	90.0

Marital Status	Currently married	3678	54.2
	Formerly married	232	3.4
	Never married	2882	42.4

Ever attended school	Yes	6489	95.6
	No	302	4.4

Area of residence	Urban	1293	19.0
	Rural	5498	81.0

Region	North	785	11.6
	Central	3201	47.1
	South	2805	41.3

Ever used the internet	Yes	1261	18.6
	No	5530	81.4

Do you listen to the radio	Yes	5728	84.4
	No	1063	15.6

Ever been tested for HIV	Yes	5255	81.4
	No	1536	22.6

### 3.1. Factors Associated With the Use of HIVST

As depicted in Table 2, the Chi‐square test found that location (*p* < 0.001), region (*p* < 0.001), ever been tested (*p* < 0.001), and using the internet (*p* < 0.001) were associated with the utilization of HIVST among men. However, listening to the radio (*p* = 0.232), marital status (*p* = 289), and ever attended school (*p* < 0.07)) were not statistically significantly associated with HIVST.

**Table 2:** Factors associated with the use of hiv self‐test among men.

Variable	Category	Ever used self test		*p* value
		Yes *n*, (%)	No *n*, (%)	
Location of residence	Rural	460 (8.4%)	5039 (91.6%)	< 0.001
	Urban	217 (16.8%)	1076 (83.2%)	

Region	North	35 (4.5%)	750 (95.5%)	< 0.001
	Central	173 (5.4%)	3028 (94.6%)	
	Southern	468 (16.7%)	2337 (83.3%)	

Listen to the radio	Yes	581 (10.1%)	5148 (89.9%)	0.232
	No	95 (8.9%)	967 (91.1%)	

Used internet	Yes	221 (17.5%)	1040 (82.5%)	< 0.001
	No	456 (8.2%)	5074 (91.8%)	

Ever attended school	Yes	656 (10.1%)	5833 (89.9%)	< 0.071
	No	21 (6.9%)	282 (93.1%)	

Ever been tested	Yes	644 (91.3%)	4610 (87.7%)	< 0.001
	No	32 (2.1%)	1505 (97.9%)	

Marital status	Currently married	382 (10.4%)	3295 (89.6%)	0.289
	Formerly married	18 (7.8%)	214 (92.2%)	
	Never married	276 (9.6%)	2606 (90.4%)	

### 3.2. Factors Affecting the Utilization of HIV Self‐Testing

The random effects model indicated that an interclass correlation coefficient of 33% indicating that 33% of the variance in the dependent variable is due to group differences, while 67% is due to individual‐level differences. The ICC dropped to 27% after adding predictor variables, indicating a much larger variance in the dependent variable attributable to individual differences. In general, the unconditional probability of a male using HIVST is 93.1%. The fixed effects model showed that ever using the internet, ever attending school, region, and urban residence were significant contributors (*p* < 0.05). The model indicated that ever used the internet was significantly associated with HIVST among men (OR = 1.535, CI: [1.231, 1.9150], *p* < 0.05). This indicates that 1 unit change in the predictor leads to 53.5% in the outcome. Moreover, the model showed that ever tested for HIV was significantly associated with lower odds of using HIVST among men in Malawi (OR = 0.181, CI: [0.122, 0.268], *p* < 0.001). An increase of 1 unit of attending school led to a decrease of 82% in the odds of utilizing HIVST among men. Rural residence was significantly associated with lower odds of utilizing HIVST among men (OR = 0.493, CI: [0.349, 0.698], *p* < 0.001). The southern region was significantly associated with higher odds of utilizing HIVST (OR = 3.619, CI: [2.120, 6.178], *p* < 0.001), indicating that an increase in the predictor led to 3.5 times higher odds of utilizing HIVST (Table 3).

**Table 3:** Fixed and random effects of the factors influencing the utilization of HIV self‐test among Malawian males.

Covariates	Model 1 (OR, CI)	Model 2 (OR, 95% CI)
Ever used the internet		
Yes		1.535^∗^ (1.231, 1.9150)
No		0

Listening to radio		

Ever attended school		
Yes		1.240 (0.727,2.115)
No		0

Ever tested for HIV		
Yes		0.181^∗^ (0.122, 0.268)
No		0

Area of residence		
Urban		0
Rural		0.493^∗^ (0.349, 0.698)

Region		
North		0
Central		0.966 (0.550, 1.695)
South		3.619^∗^ (2.120, 6.178)

Marital Status		
Currently Married		0
Formerly Married		0.762 (0.443, 1.313)
Never Married		1.159 (0.947, 1.417)

ICC	33.8%	27.6%

Unweighted *N*	7750	7750

Weighted *N*	6575	6575

*Note:* 95% confidence intervals (CI) in brackets, intracluster correlation coefficient (ICC), N number of observations, and 0.00 = reference.

Abbreviation: OR, odds ratio.

^∗^ *p* < 0.05.

## 4. Discussion

In this study, ever used the internet, ever tested for HIV, area of residence, and region of residence were significant predictors of using HIVST among men. On the other hand, marital status and ever attended school were not significant predictors in other use of HIVST among men. Community‐level factors had a strong effect on the utilization of HIVST compared to individual‐level factors.

In this review, only 10% of men have ever used an HIVST. The finding of the study is higher than what was reported in the neighboring Tanzania [3]. In addition, lower rates were also reported in a study conducted in South Africa [15]. The findings of this study are lower than the rates by Hong [16], who reported 38.2% of men who used HIVST kits. The reasons for the relatively low HIVST rate in our study could be attributed to the difference in the methodological approach, as the current study utilized a national survey representing a broad geographical area in both urban and rural areas in Malawi, contrary to the study by Hong et al. (2021) in China, which focused on data from key populations, potentially more aware of and willing to use HIV self‐testing. Additionally, the low rates of uptake could also be related to the lack of interventions to promote HIV self‐testing. The study finding calls for increased awareness programs to improve the utilization of HIVST among men in the country.

In this paper, there was a strong and statistically significant association between region and HIV self‐testing among Malawian men, with the southern region showing notably higher uptake (12.26%) compared to the central (4.30%) and northern (3.85%) regions (*p* < 0.001). This finding is not unexpected considering the context of the study. According to a recent study published in 2025, HIV testing in sub‐Saharan Africa varies with geographical settings [17]. Similarly, in Malawi, healthcare services are faced with regional disparities [18]. This clear regional disparity suggests that broader access to services, better health communication, and possibly greater normalization of self‐testing in the south are key drivers, reinforcing that geography remains a critical determinant of health behaviors. Therefore, this finding highlights the need to prioritize the underprivileged areas to improve access to services.

In this study, the type of residence was also found to be a significant attribute to the use of self‐test kits. This is in agreement with the research by Aloni [3], who reported that men residing in rural areas were significantly 33% less likely to test their HIV status using the HIVST kits as compared to those living in urban areas. The findings of our study can be explained by the availability of health information in urban settings through social media platforms and other mass media communication tools, which are more widely accessible in urban than rural areas. The advent of e‐health has opened numerous opportunities for health communication and promotion by providing ease of client and practitioner engagement and interactions, including the sharing of medical information among patients and the general public with ease and cost‐effectiveness [19]. The study conducted by Yaya and Ghose [19] in more than four countries, including Malawi, found that access to computers and the internet varied depending on the place of residence.

In this paper, HIV self‐testing was also significantly associated with internet use. The finding is surprising considering that in Malawi use of Information Communication Technology (ICT) in health services remains challenged by the high cost of internet bundles and internet devices as well as a lack of support from top management at the Ministry of Health offices [20]. The strong link highlights how digital exposure can empower men with confidential health information and promote proactive health decisions, suggesting that internet access is not just a technological advantage but a public health catalyst in expanding HIV self‐testing uptake. Therefore, the study calls for government and relevant stakeholders to ensure support to improve the ICT landscape in the country to ensure improved access to HIVST.

The findings of this study show that ever attending school is not a significant predictor of using HIVST kits. The findings are inconsistent with Aloni [3] and Myres et al. [21], who found that the usage of HIVST kits was significantly higher among more educated men than those with primary education or less. These positive relationships may have been attributed to several factors, including educated people having better access to health information, which resulted in an understanding of the importance of testing for HIV using medical tools such as HIVST kits. The differences among the studies could be attributed to different contextual factors among countries.

## 5. Limitations

The study is not without limitations. First, the study does not provide an in‐depth understanding of the issue due to the quantitative nature of the study design. In addition, the study does not fully explain the health belief model since other constructs of the model related to perception and self‐efficacy were not assessed. Moreover, the findings are prone to recall bias due to the subjective nature of the data collection method of the survey. Nevertheless, the study is the first of its kind to unravel this phenomenon and provide a baseline for future studies on the topic. Furthermore, the multiple logistic regression is more reliable in terms of odds and generalization to the entire population.

## 6. Conclusion

In conclusion, the study has shown that there is low uptake of HIVSTs among men in Malawi, calling for concerted efforts to raise awareness about HIVSTs and promote uptake, and ultimately contribute to the achievement of the 90‐90‐90 goal by 2030. Furthermore, the study has unveiled that the use of HIVSTs among men in Malawi is characterized by a socioeconomic divide entrenched in location of stay, region, and access to the internet, among other things. Efforts to improve uptake of self‐tests among men should consider this socioeconomic disparity to ensure that the most vulnerable are reached and ensure universal health coverage. Further studies can explore the challenges and opportunities of men using HIVSTs to explore an in‐depth analysis of the phenomenon. Moreover, further research can employ discreet choice experiments to elicit men’s preferences for HIVST.

## Ethics Statement

Ethical approval was waived due to the nature of the condition. Permission to use the data was obtained from UNICEF.

## Conflicts of Interest

The authors declare no conflicts of interest.

## Funding

The authors received no financial support for the research, authorship, and/or publication of this article.

## Data Availability

The data are available upon request via the following link: https://mics.unicef.org/surveys.
